# Thank you to our reviewers in 2015

**DOI:** 10.1051/sicotj/2016001

**Published:** 2016-02-02

**Authors:** Jacques Caton, Hatem Galal Said

**Affiliations:** 1 Clinique Orthopédique Emilia de Vialar Lyon France; 2 Assiut University Hospital Assiut Egypt

The editorial team would like to acknowledge all the reviewers that have accepted and completed a review in 2015 for SICOT-J. With their valuable and constructive comments they have contributed to the quality of the articles published in SICOT-J. The journal relies on their personal effort, sharing their expertise and input and is therefore very grateful.

Amr Abdel Hady

Nasef Mohamed Nasef Abdelatif

Hisham Abdel-Ghani

Ashraf Abdelkafy

Mohamed El-Sayed Abdel-Wanis

Nariman Abol Oyoun

Ijaz Ahmad

alaa azmi ahmad

Khaled Al Saleh

Nuri Aydin

Ufuk Aydinli

Ahmed Azeem

Laszlo Bucsi

Jacques Caton

Hitendra Doshi

Ziyad El Qirem

Ossama El Shazly

Yasser Elbatrawy

Mohammad El-Sharkawi

Essam ElSherif

Khaled Emara

Mehmet Erdil

Osama Farouk

André Ferreira

Matt Fletcher

Patricia Fucs

Boris Holzapfel

Ashok Johari

Ahmed Kandil

Sherif Khaled

Ahmed Khater

Wael Koptan

Fatih Kucukdurmaz

Anna Kulidjian

Ernest Kwek

Ian Lesley

Noppachart Limpaphayom

François Loisel

Keith Luk

Sébastien Lustig

Mostafa Mahmoud

Mahmoud Mahran

Oliver Marin Pena

Robert Meves

Ashraf Moharram

Jean-Louis Prudhon

Narayan Ramachandran

Maximilian Rudert

Waleed Riad Saleh

Alpaslan Senkoylu

Rajasekaran Shanmuganathan

Vijay Shetty

Mohamed H Sobhy

